# The effect of nuciferine on the renal dysfunction following ischemia-reperfusion injury

**DOI:** 10.1371/journal.pone.0320320

**Published:** 2025-03-26

**Authors:** Sundus M. Sallabi, Loay Lubbad, Harun R. Toumi, Waheed F. Hammad, Manjusha Sudhadevi, Jasmine Abdul Rasheed, Awwab F. Hammad, Mustafa M. Ardah, Suhail Al-Salam, Fayez T. Hammad

**Affiliations:** 1 Department of Surgery, College of Medicine & Health Sciences, United Arab Emirates University, Al Ain, United Arab Emirates; 2 School of Medicine, University of Jordan, Amman, Jordan; 3 Department of Pathology, College of Medicine & Health Sciences, United Arab Emirates University, Al Ain, United Arab Emirates; 4 Department of Biochemistry and Molecular Biology, College of Medicine & Health Sciences, United Arab Emirates University, Al Ain, United Arab Emirates; Osaka University of Pharmaceutical Sciences, JAPAN

## Abstract

Renal ischemia-reperfusion injury (IRI) is an inevitable consequence of several clinical conditions and surgical procedures including renal transplantation and resuscitation following systemic hypotension. It leads to immediate renal dysfunction and might result in long-term renal damage. Therefore, there is an ongoing effort to mitigate the deleterious effects of the IRI on the kidney. Recently, there has been a recent interest in using natural compounds as alternative remedies in many diseases. Thus, the aim of this research was to study the effect of nuciferine, a phytochemical compound extracted from the plant *Nelumbo nucifera Gaertn,* on the renal dysfunction in a rat mode of IRI. Nuciferine was administered orally as single daily dose of 30 mg/kg for 9 days prior to IRI and continued for 3 days post-IRI. G-Sham (n = 11) underwent sham surgery whereas G-IRI (n = 12) and G-IRI/NF (n = 12) underwent bilateral warm renal ischemia for 35 minutes. G-IRI/NF received nuciferine. Renal functions and histological changes were assessed before starting the medication, just prior to IRI and 3 days after IRI. Nuciferine significantly attenuated the alterations in serum creatinine, serum urea, creatinine clearance and urinary albumin creatinine ratio. This was associated with significant attenuation of the alterations in renal injury markers, several cytokines including pro-inflammatory, pro-fibrotic and apoptotic cytokines and in histological changes. In conclusion, nuciferine has reno-protective effects on the IRI-induced renal dysfunction. These findings might be of clinical significance.

## Introduction

Renal ischemia-reperfusion injury (IRI) is commonly encountered in several clinical conditions and surgical procedures such as kidney transplantation, partial nephrectomy and resuscitation following systemic hypotension [1]. Ischemia-reperfusion injury leads to several pathophysiological responses and alterations such as oxidative stress, pro-inflammatory, pro-apoptotic and pro-fibrotic responses [2–6] which, in turn, result in alternations in glomerular and renal tubular functions [7]. Due to its deleterious effects on the kidney, there is an ongoing search for therapeutic agents that can effectively reduce the renal effects of this condition [3–6,8,9].

The use of phytochemical compounds in treating renal IRI has been well-documented in the literature [3,5,10–12]. Their use has been encouraged by the wide availability and relatively low toxicity. For instance, berberine, an alkaloid, has been shown to have a protective effect on the kidney following IRI as demonstrated by attenuating the alterations in the renal functional parameters such as serum creatinine and blood urea nitrogen associated with down-regulation of acute renal injury markers and other inflammatory cytokines [10]. Similarly, nerolidol, a naturally occurring sesquiterpene alcohol, exhibited anti-inflammatory, antioxidant, and anti-apoptotic effects against renal IRI, resulting in the mitigation of the alterations in the key renal functional parameters including serum creatinine, creatinine clearance, and the albumin creatinine ratio [3]. These findings suggest that phytochemical compounds hold significant potential as protective agents against renal IRI.

Nuciferine (NF) is a naturally occurring aporphine-type alkaloid which was first isolated from the leaves of *Nelumbo nucifera* Gaertn [13] and subsequently extracted from other plants such as *Michelia champaca* L [14]. It has been shown to have a variety of important biological properties including anti-inflammatory, anti-viral, antioxidant, anti-tumor and anti-diabetic activities [15]. For instance, nuciferine was found to have an anti-diabetic effect as it stimulated insulin secretion from beta cells by closing the potassium-adenosine triphosphate channels [16]. It has also been shown to attenuate the isoproterenol-induced myocardial infarction in rats [17]. In addition, nuciferine inhibited cutaneous melanoma cell growth and tumor size by repressing the phosphorylation of p65 and TLR4/NF-κB signaling pathway [18]. Moreover, it has mitigated the inflammatory response associated with lipopolysaccharide-induced acute lung injury through repressing the expression of TLR4 and the activation of NF-κB [19].

In the kidney, very few studies have shown a protective effect of nuciferine. For example, nuciferine has been shown to be protective against folic acid-induced acute kidney injury by inhibiting ferroptosis [20]. It also restored the potassium oxonate-induced hyperuricemia and kidney inflammation [21]. Further, nuciferine has alleviated the fructose-induced renal Injury by inhibiting the inflammatory responses [11]. However, the effect of nuciferine on the renal dysfunction following IRI has not been studied yet and hence the aim of this study was to investigate this effect in a rat model of bilateral warm renal IRI.

## Materials and methods

Male Wistar rats weighing around 200 g, were used in the experiments. A standard rat chow was used to feed the animals which were fasted for 12 hours before the experimental procedures but had water *ad libitum*. The experimental protocol was approved by the local ethics committee (ERA_2023_2736).

### Ischemia-reperfusion injury

All surgical procedures were carried out under strict aseptic conditions as previously described [3,4]. In summary, after anesthetizing the rats using intraperitoneal injection of pentobarbitone (50 mg/kg), the right and left renal arteries were exposed and dissected. Ischemia was created by applying microsurgical non-traumatic bulldog clamps which were applied simultaneously on both arteries for 35 minutes. Reperfusion was restored by releasing the clamps. The surgical incision was then closed in layers.

### Experimental protocol and nuciferine and vehicle administration

Nuciferine (MuseChem, New Jersey, USA) was dissolved in 0.5ml of the vehicle which is composed of carboxymethyl cellulose 0.5%, Tween-80 1.0% and polyethylene glycol-400 1.0% in distilled water and administered by oral gavage immediately after preparation as a single daily dose of 30 mg/kg. This dose was chosen based on the findings of previous studies that used similar doses which had protective effect in other conditions [11,20,22]. Using similar oral doses, nuciferine was shown to have a reasonable bioavailability [23] with the highest concentration found in the kidney and lungs followed by other organs [23,24]. The LD_50_ of orally administered nuciferine in rats was found to be 280 mg/kg [25]. The vehicle composed of substances which were shown to either improve the solubility and bioavailability of the drug or to make it easier to handle and administer via the gavage needle [26–29].

**Fig 1:**
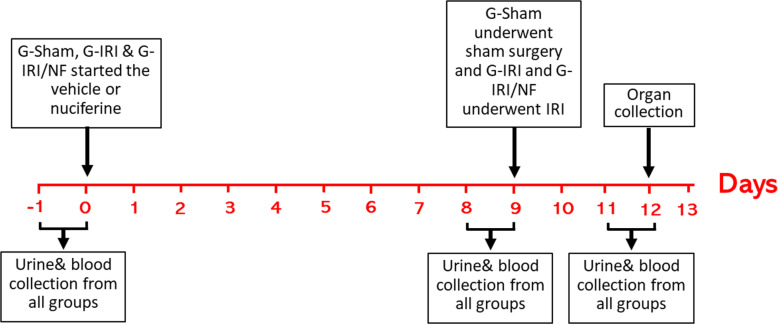
Schematic presentation of the study plan showing interventions in all the three groups.

As shown in Fig 1, the treatment started 9 days before the renal IRI/Sham surgery and continued for 3 days after the procedure. None of the treated animals showed any adverse effect and there was no mortality reported.

### Experimental groups

The rats were assigned randomly to three groups:

G-Sham (n = 11): Rats which underwent sham manipulation of both renal arteries and received the vehicle.
G-IRI (n = 12): Rats which underwent warm bilateral renal ischemia for 35 minutes and received only the vehicle.
G-IRI/NF (n = 12): Rats which underwent bilateral renal ischemia for 35 minutes and received nuciferine dissolved in the vehicle.

### Sample collection and analysis

Metabolic cages were used to collect urine for 24 hours at three different time points: just before the start of nuciferine/vehicle treatment for the measurement of baseline pre-treatment values (Basal), day 9 after nuciferine/vehicle treatment and before IRI/Sham procedure (Pre-IRI) and on the third day after IRI (Post-IRI) (Fig 1). Using the tail vein, blood was also withdrawn from the tail vein at the same time of urine collection. The samples were frozen at -30^0^C for the measurement of urea, albumin and creatinine levels. Seventy-two hours post-IRI/Sham procedure, the animals were anesthetized using intraperitoneal injection of pentobarbitone (50 mg/kg) and the kidneys were collected and stored in either liquid nitrogen then at -80^0^C or in formalin for further assays. The animals were sacrificed by removal of the heart under anesthesia after obtaining the tissues needed for the experiment. During the experimental protocol, animals were closely monitored for signs of pain and distress to deliver subcutaneous analgesia (Butorphanol 2mg/kg), albeit no animals required this.

### Gene expression analysis

A wedge from the middle part of the left kidney which contained both cortex and medulla was obtained and snap-frozen in liquid nitrogen. It was then stored at -80°C for later measurement of gene expression of the following by reverse transcription polymerase chain reaction (RT-PCR):

1. Acute kidney injury markers: Kidney injury molecule-1 (KIM1) and neutrophil gelatinase-associated lipocalin (NGAL).
2. Cytokines involved in inflammation and fibrosis: Interlukin-1 beta (IL-1β), tumor necrosis factor-α (TNF-α), moncyte chemoattractant protein-1 (MCP-1), transforming growth factor-β (TGF-β1) and plasminogen activator inhibitor-1 (PAI-1).
3. The pro-apoptotic gene p53.
4. Procollagen type-1 (COLA-1).

Extraction of the total RNA from the frozen samples was performed using Qiazol Lysis reagent (Qiagen, Hilden, Germany) as previously described [3,4,30]. In summary, the estimation of the quantity and quality of the extracted RNA was performed using NanoDrop 2000 Spectrophotometer (Thermo Fisher Scientific Inc, Wilmington, Delaware USA).

Preparation of the strand complementary DNA (cDNA) in duplicates from 1.0 µg of extracted RNA was achieved using QuantiTect® reverse transcription kit (Qiagen, Hilden, Germany). The genomic DNA was removed using the supplied gDNA wipeout buffer. The prepared cDNA was then used as a template for relative gene expression analysis using the Taqman® hydrolysis probe chemistry. The probes were FAM-labeled. Peptidylprolyl Isomerase A (PPIA) house-keeping gene was used for normalization. Its probe was labeled with Quasar 670. Table 1 shows the sequences of primers and probes. The calculated Cycle Threshold (CT) values were used to estimate the changes in gene expression of target genes using delta–delta CT formula. The cycle threshold (CT) values were used to estimate the delta-delta CT(ΔΔCT) to determine changes in the expression of the target genes.

**Table 1:** Forward and reverse primers and fluorogenic probe sequences used for real time quantitative PCR analysis. KIM-1: kidney injury molecule-1; NGAL: neutrophil gelatinase-associated lipocalin also called lipocalin 2 (Lcn2); TNF-α: tumour necrosis factor-alpha; TGF-β1: transforming growth factor-β; IL-1β: interleukin 1 beta; PAI-1: plasminogen activator inhibitor-1; MCP-1: moncyte chemoattractant protein-1; p53: pro-apoptotic gene p53; COLA-1: procollagen type-1; PPIA: peptidylprolyl isomerase A (housekeeping gene).

KIM-1 (NM_173149.2)	Forward	GCCTGGAATAATCACACTGTAAG
Reverse	GCAACGGACATGCCAACATAG
Probe	d FAM-TCCCTTTGAGGAAGCCGCAGA-BHQ-1
Lipocalin 2 (LCN2) (NM_130741.1)	Forward	CTGTTCCCACCGACCAATGC
	Reverse	CCACTGCACATCCCAGTCA
	Probe	FAM-TGACAACTGAACAGACGGTGAGCG-BHQ-1
TNF-α (NM_012675.3)	Forward	CTCACACTCAGATCATCTTCTC
	Reverse	CCGCTTGGTGGTTTGCTAC
	Probe	FAM-CTCGAGTGACAAGCCCGTAGCC-BHQ-1
TGF-β1 NM_012620.1	Forward	GTGGCTGAACCAAGGAGACG
	Reverse	CGTGGAGTACATTATCTTTGCTGTC
	Probe	FAM-ACAGGGCTTTCGCTTCAGTGCTC-BHQ-1
PAI-1 (NM_012620.1)	Forward	GGCACAATCCAACAGAGACAA
	Reverse	GGCTTCTCATCCCACTCTCAAG
	Probe	FAM-CCTCTTCATGGGCCAGCTGATGG-BHQ-1
MCP-1 (NM_031530.1)	Forward Primer	GCTGTCTCAGCCAGATGCAG
	Reverse Primer	CCAGCCGACTCATTGGGA
	Probe	FAM-CCCACTCACCTGCTGCTACTCA-BHQ-1
IL-1β (NM_031512.2)	Forward	ATGCCTCGTGCTGTCTGACC
	Reverse	GCTCATGGAGAATACCACTTGTTGG
	Probe	FAM-AGCTGAAAGCTCTCCACCTCAATGGA-BHQ-1
p53 (NM_030989.3)	Forward	CGAGATGTTCCGAGAGCTGAATG
	Reverse	GTCTTCGGGTAGCTGGAGTG
	Probe	FAM-CCTTGGAATTAAAGGATGCCCGTGC-BHQ-1
COLA-1 (NM_053304.1)	Forward	CTGACTGGAAGAGCGGAGAGT
	Reverse	CCTGTCTCCATGTTGCAGTAGAC
	Probe	FAM-ACTGGATCGACCCTAACCAAGGC-BHQ-1
PPIA (NM_017101.1)	Forward	GCGTCTGCTTCGAGCTGT
	Reverse	CACCCTGGCACATGAATCC
	Probe	Quasar 670-TGCAGACAAAGTTCCAAAGACAGCA-BHQ-2

### Western blot analysis

The proteins were extracted from the frozen kidney samples using freshly made 1X RIPA buffer (0.05 M Tris-HCl (pH 7.4), 0.15 M NaCl, 1 mM Na_2_EDTA, 1% Igepal, 0.25% sodium deoxycholate, 1μg/ml proteinase inhibitor cocktail). The extracted proteins (15-30 g) were diluted in 5X loading buffer and loaded onto 12% SDS-PAGE gels. The proteins were then transferred onto a polyvinylidene difluoride (PVDF) membrane and blocked with 5% non-fat milk diluted in PBS-T (0.5% Tween-20). The membranes were incubated overnight at 4 °C with the following primary primary antibodies: anti-p-NF-κB p65 (1:1000, CAT# 3033S, Cell Signaling Technology® (CST), anti-cleaved caspase-3 (1:1000, CAT# 9664S, CST), anti-LC3 (1:1000, CAT# 12741S, CST), anti-IL-18 (1/5000, CAT# 10663-1-AP, Protein Tech), and GAPDH (1:1000, CAT # 2118S, CST). All blots were then incubated at room temperature with diluted goat anti-rabbit secondary antibodies conjugated with horseradish peroxidase enzyme (1:20000). The bands were visualized using a chemiluminescence West Pico kit, and signals were detected using X-ray film. ImageJ software (NIH, Milwaukee, WI, USA) was used for the quantification of the bands.

### Histological studies

The kidney tissues were washed with ice-cold saline and blotted using filter paper, cassetted and fixed directly in 10% neutral buffered formalin for 24 hours. They were then dehydrated using increasing ethanol concentrations, cleared, and embedded in paraffin. This was followed by preparation of 3 μm sections from paraffin blocks which was stained with hematoxylin and eosin. The stained sections were evaluated blindly using light microscopy.

The microscopic scoring was performed by measuring the percentage of the areas which showed morphologic changes (tubular dilatation, tubular atrophy, interstitial fibrosis, and mononuclear cellular infiltrate) in comparison to the total surface area in the field. Measurement of the frequency of each histological abnormality was performed using Image J software (NIH, USA).

### Statistical analysis

Statistical analysis was performed using SPSS V16.0. Results were expressed as mean±SEM. One-way factorial ANOVA was used for comparison of variables between groups and between different stages (Basal, Pre-IRI and Post-IRI) within each group. P value less than 0.05 was considered statistically significant.

## Results

As demonstrated in Table 2, the Basal and Pre-IRI values of serum creatinine, serum urea, creatinine clearance, 24-hour urinary albumin and albumin/creatinine ratio (ACR) were similar in all the groups (P > 0.05 for all variables). Likewise, none of the groups had shown any difference in any variable between the Pre-IRI and Basal values (P > 0.05 for all variables).

**Table 2:** Renal functional parameters in all the three groups (G-Sham, G-IRI and G-IRI/NF) at different stages in the experimental protocol; Basal: before the start of treatment; Pre-IRI: just prior to the intervention (IRI or sham surgery) and post-treatment with nuciferine or vehicle; Post-IRI: 72 hours after the IRI or sham surgery. Results represent mean ±  SEM.

	G-Sham			G-IRI			G-IRI/NF		
	**Basal**	**Pre-IRI**	**Post-IRI**	**Basal**	**Pre-IRI**	**Post-IRI**	**Basal**	**Pre-IRI**	**Post-IRI**
**Serum Creatinine (mg/dl)**	**0.22 ± 0.01**	**0.20 ± 0.01**	**0.21 ± 0.01**	**0.22 ± 0.01**	**0.22 ± 0.01**	**0.64 ± 0.14** ^$^	**0.21 ± 0.01**	**0.25 ± 0.01**	**0.37 ± 0.02** ^*^
**Serum Urea (mg/dl)**	**24.9 ± 0.4**	**24.5 ± 0.8**	**23.4 ± 0.7**	**25.3 ± 0.8**	**25.6 ± 0.8**	**85.4 ± 21.5** ^$^	**27.1 ± 0.9**	**25.1 ± 0.8**	**37.3 ± 1.8** ^*^
**Creatinine Clearance (ml/min/100g b. w.)**	**1.00 ± 0.06**	**1.06 ± 0.05**	**1.01 ± 0.03**	**1.02 ± 0.05**	**0.99 ± 0.05**	**0.44 ± 0.08** ^$^	**1.04 ± 0.08**	**0.99 ± 0.07**	**0.67 ± 0.06** ^*^
**24-hour Urinary Albumin (**µ)	**6.79 ± 0.80**	**6.88 ± 1.01**	**7.53 ± 1.13**	**6.85 ± 0.37**	**7.80 ± 0.99**	**436.78 ± 67.49** ^$^	**7.50 ± 1.58**	**8.90 ± 1.59**	**249.44 ± 28.53** ^*^
**Albumin Creatinine Ratio**	**12.4 ± 1.7**	**12.0 ± 3.0**	**9.2 ± 1.2**	**11.4 ± 1.2**	**10.2 ± 1.7**	**209.4 ± 27.3** ^$^	**12.3 ± 1.9**	**11.4 ± 1.9**	**138.4 ± 14.6** ^*^

$ indicates statistical significance when compared to the Sham group (G-Sham).

*Indicates statistical significance when compared to the G-IRI group.

In the G-Sham group, manipulation of renal arteries did not affect any variable when compared to the Pre-IRI value (P > 0.05 for all variables). In the G-IRI, there was significant alterations in the renal functions after IRI (Table 2). For instance, IRI led to a significant deterioration in serum creatinine and creatinine clearance (0.64 ± 0.14 vs. 0.22 ± 0.01 and 0.44 ± 0.08 vs. 0.99 ± 0.05, respectively, P < 0.05 for both). Likewise, IRI led to an increase in the urinary albumin leak. So, the ACR has increased to 209.4 ± 27.3 vs. 10.2 ± 1.7, P < 0-001.

As shown in Table 2, the administration of nuciferine has significantly attenuated the IRI-induced alterations in all these parameters (P < 0.05 for all variables).

### Gene expression analysis

**Fig 2:**
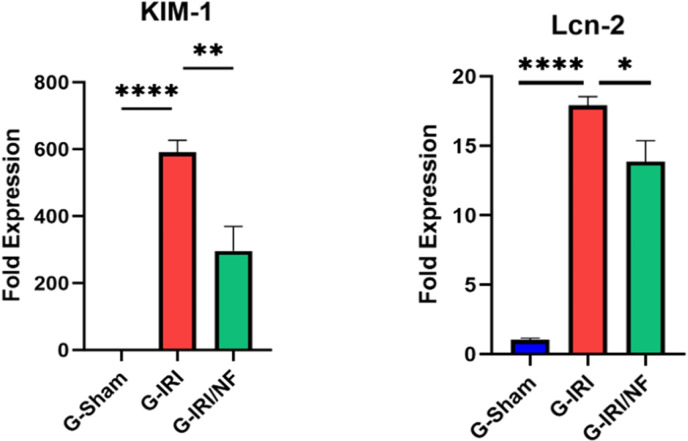
The gene expression of two of markers of acute renal injury in the three groups 72 hours post-ischemia or sham surgery; (KIM-1: Kidney injury molecule-1 and NGAL: neutrophil gelatinase-associated lipocalin) in all the three groups. Values represent mean±SEM. *  P < 0.05, ** P < 0.01, **** P < 0.0001.

As illustrated in Fig 2, IRI caused a significant increase in the gene expression of KIM-1 and NGAL compared to G-Sham (590.0 ± 36.7 vs. 1.1 ± 0.3 and 17.9 ± 0.6 vs. 1.0 ± 0.1, respectively, P < 0.001 for both). Nuciferine treatment has significantly attenuated this increase (296.2 ± 73.5 vs. 590.0 ± 36.7 (P < 0.01) and 13.8 ± 1.5 vs. 17.9 ± 0.6 (P < 0.05), respectively.

**Fig 3:**
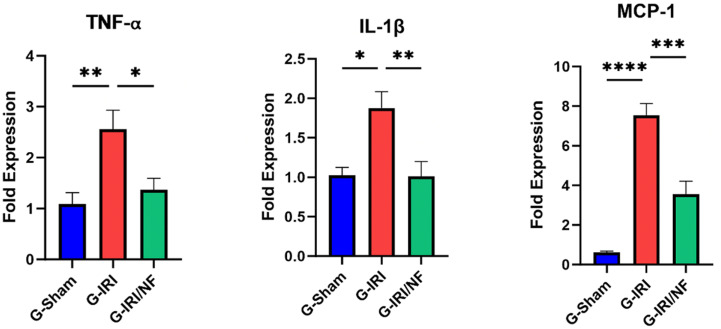
The gene expression of pro-inflammatory markers: tumor necrosis factor- α (TNF-α), interleukin 1 beta (IL-1β) and moncyte chemoattractant protein-1 (MCP-1) in all the three groups 72 hours post-ischemia or sham surgery. Values represent mean±SEM. *  P < 0.05, ** P < 0.01, *** P < 0.001, **** P < 0.0001.

**Fig 4:**
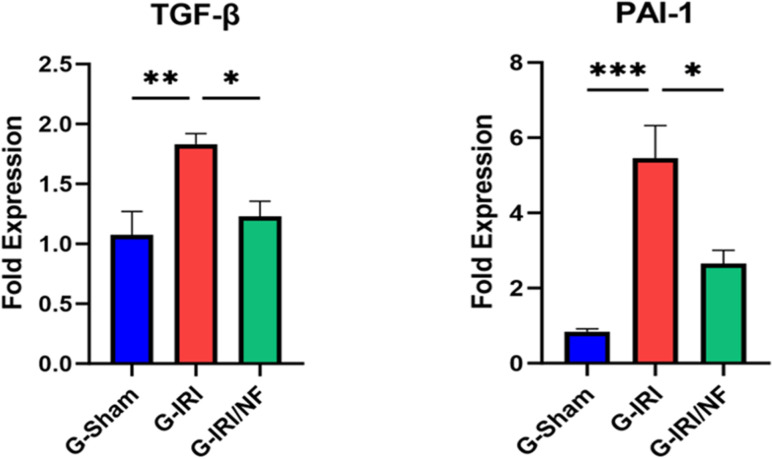
The gene expression of the pro-fibrotic markers: transforming growth factor- β (TGF-β1) and plasminogen activator inhibitor-1 (PAI-1) in all the three groups 72 hours post-ischemia or sham surgery. Values represent mean±SEM. *  P < 0.05, ** P < 0.01, *** P < 0.001.

**Fig 5:**
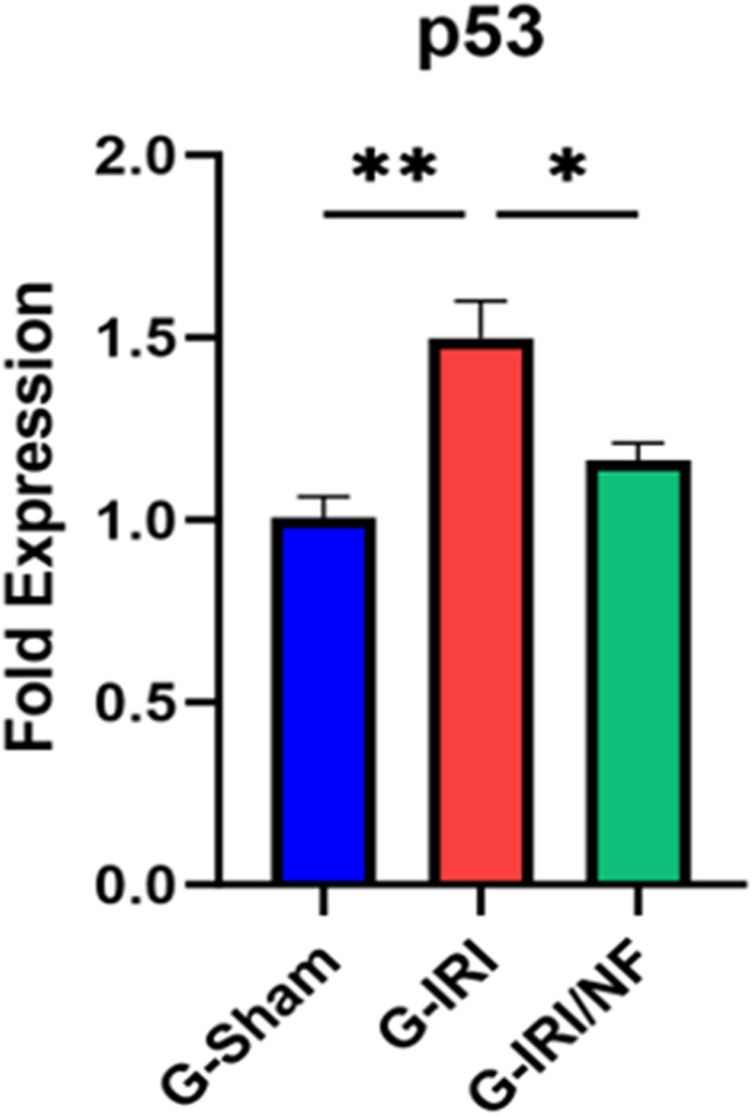
The gene expression of the pro-apoptotic p53 gene in all the three groups 72 hours post-ischemia or sham surgery. Values represent mean±SEM. *  P < 0.05, ** P < 0.01.

A similar trend was observed in relation to pro-inflammatory, pro-fibrotic and pro-apoptotic cytokines (Fig 3–4 and Fig 5). For example, the mRNA expression of IL-1β, TGF-β and p53 was significantly upregulated in G-IRI compared with G-Sham (1.88 ± 0.24 vs. 1.02 ± 0.10, P < 0.05), (1.83 ± 0.09 vs. 0.97 ± 0.08, P < 0.01) and (1.49 ± 0.10 vs. 0.96 ± 0.04, P < 0.01), respectively, while NF treatment significantly downregulated their expression when comparing G-IRI/NF with G-IRI (1.01 ± 0.18 vs. 1.88 ± 0.24, P < 0.01), (1.23 ± 0.13 vs. 1.83 ± 0.09, P < 0.05) and (1.16 ± 0.05 vs. 1.49 ± 0.08, P < 0.05), respectively. Similar effects were observed in the gene expression of TNF-α (P < 0.05), MCP-1 (P < 0.001) and PAI-1 (P < 0.05).

**Fig 6:**
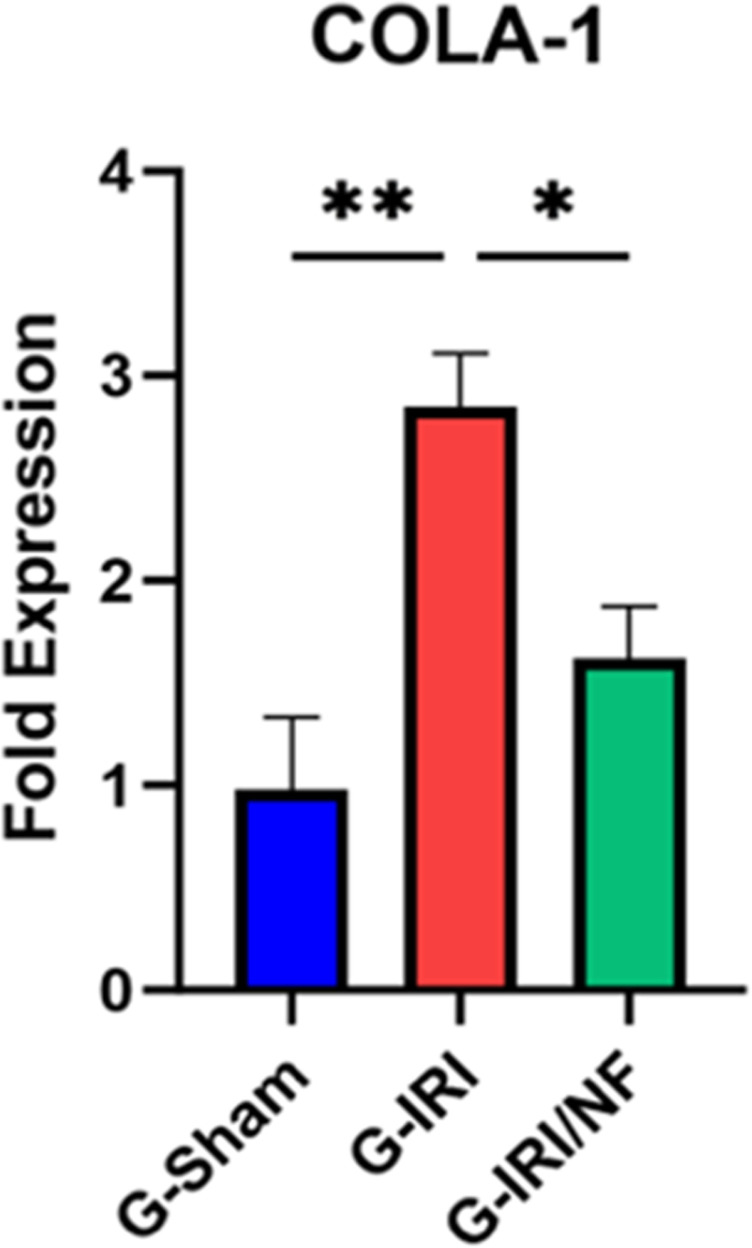
The gene expression of procollagen-1 (COLA-1) in all the three groups 72 hours post-ischemia or sham surgery. Values represent mean±SEM. *  P < 0.05, ** P < 0.01.

The gene expression of procollagen type-1 (COLA-1) has also increased significantly with IRI (2.85 ± 0.26 vs. 0.98 ± 0.02, P < 0.01) and NF treatment has ameliorated this alteration (1.62 ± 0.26 vs. 2.85 ± 0.26, P < 0.01) (Fig 6).

### Western blot analysis

**Fig 7:**
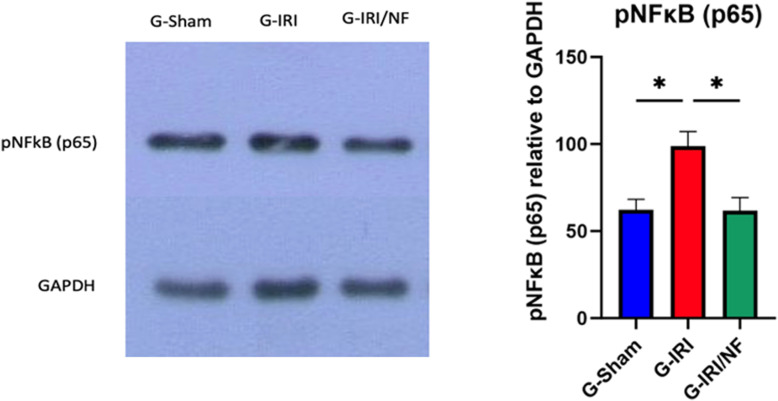
Protein expression of pNF- κB p65 in all the three groups 72 hours post-ischemia or sham surgery. Values are expressed as the ratio of mean±SEM relative to GAPDH. *  P < 0.05.

The protein expression of the transcription factor pNF-κB p65 is shown in Fig 7. pNF-κB p65 expression markedly increased in the G-IRI compared to the G-Sham group (98.8 ± 8.4 vs. 62.2 ± 6.1, P < 0.05), while NF treatment normalized this expression (61.8 ± 7.5 vs. 98.8 ± 8.4, p < 0.05) (Fig 7).

**Fig 8:**
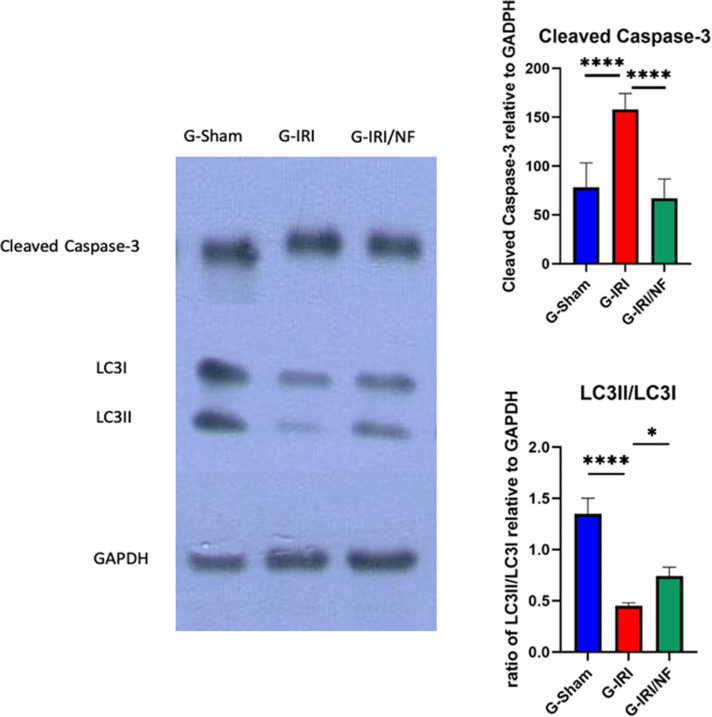
Protein expression of cleaved caspase-3 and LC3II/LC3I in all the three groups 72 hours post-ischemia or sham surgery. Values are expressed as the ration of mean ±  SEM to GAPDH. *  P < 0.05, **** P < 0.0001.

As shown in Fig 8, the expression of cleaved caspase-3 was significantly higher in the G-IRI group relative to the G-Sham group (157.8 ± 7.3 vs. 78.1 ± 10.2, P < 0.0001). This rise was mitigated by NF administration (67.1 ± 8.7 vs. 157.8 ± 7.3, P < 0.0001).

The expression of LC3II/LC3I was markedly reduced in G-IRI relative to G-Sham (0.45 ± 0.03 vs. 1.36 ± 0.15, P < 0.001), suggesting reduced autophagic flux following IRI (Fig 8). NF treatment increased autophagic flux by increasing the LC3-II/LC3-I ratio, (0.70 ± 0.08 vs. 0.45 ± 0.03, P < 0.05).

### Histological studies

**Fig 9:**
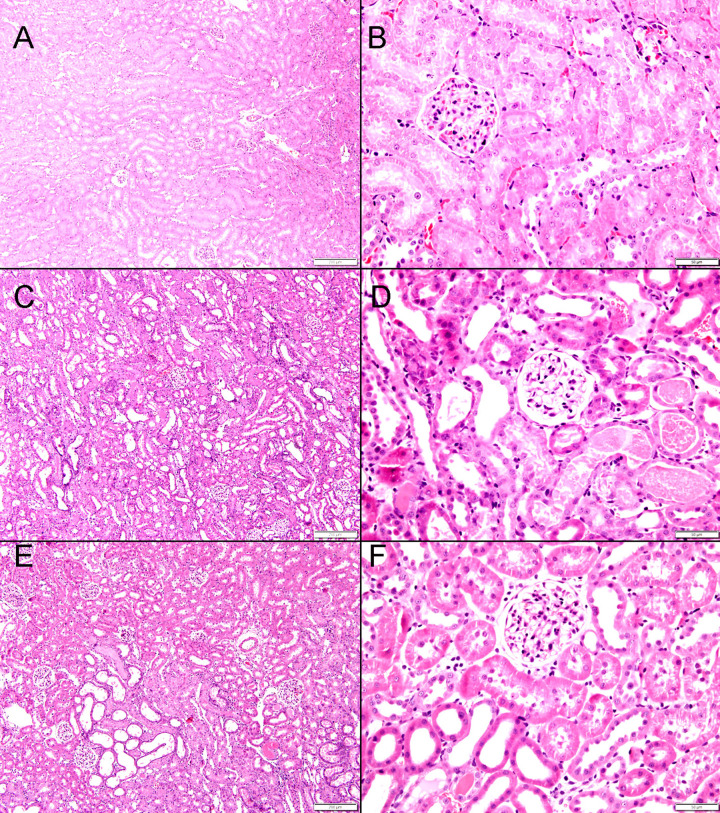
The histological features in all the experimental groups 72 hours post-ischemia or sham surgery. A& B: The kidneys in G-Sham showing normal architecture and histology; C& D: the histological features in the G-IRI with large areas of acute tubular necrosis with tubular dilatation (thin arrow) and intratubular secretions (arrowhead); E&F: Histological features in G-IRI/NR with mild acute tubular necrosis and tubular dilation (thin arrow) and intratubular secretion (arrowhead).

**Fig 10:**
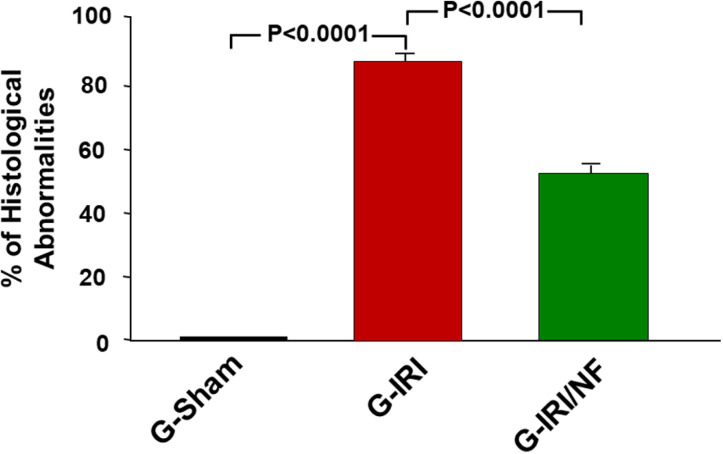
The microscopic scores in all the three groups showing the percentage of the areas which showed morphologic changes (tubular dilatation, tubular atrophy, interstitial fibrosis, and mononuclear cellular infiltrate) in comparison to the total surface area in the field, 72 hours post-ischemia or sham surgery.

Renal IRI induced significant histological changes in the architecture of the kidneys. G-Sham control group showed normal kidney architecture and histology (Fig 9 A&B). In contrast, G-IRI group showed extensive areas of acute tubular injury comprising 86.8 ± 2.2% of the examined tissue fields (Fig 10), exhibiting dilated renal tubules with intratubular eosinophilic secretion (Fig 9 C& D). Nuciferine treatment attenuated this effect and led to less extensive acute tubular injury with tubular dilatation and intratubular secretion occupying only 52.7 ± 2.7% of the fields (vs. 86.8 ± 2.2, P < 0.0001) (Fig 9 E & F and Fig 10).

## Discussion

This study is the first to investigate the effect of nuciferine on IRI-induced renal dysfunction. We have demonstrated that the administration of this phytochemical compound before and after renal ischemia has markedly ameliorated the alterations in renal functions serum creatinine, serum urea, creatinine clearance, 24-hour urinary albumin and albumin creatinine ratio. Moreover, nuciferine significantly mitigated the IRI-induced changes in the gene expression and protein levels in a variety of markers of renal injury, inflammation, fibrosis, and apoptosis. Our data also revealed a significant improvement in the renal histological features in response to nuciferine treatment.

In this study, IRI-induced upregulation of some pro-inflammatory cytokines and chemokines such as IL-1β, TNF-α and MCP-1 all of which were downregulated by nuciferine administration. The pro-inflammatory chemokine MCP-1 plays an important role in the chemoattraction of macrophages and T-cells to the site of injury [31–33]. Following the inflammatory cells chemotaxis, these cells produce pro-inflammatory cytokines, such as IL-1β and TNF-α, further mediating the activation of inflammatory cells and producing a vicious inflammatory response [32,34]. The IRI-induced alteration in these cytokines has been mitigated by nuciferine indicating a strong anti-inflammatory role against IRI-mediated renal dysfunction.

In the current study, we have shown that IRI has led to the activation of the transcription factor NF-κB as demonstrated by the increased expression and activation of pNF-κB p65 when assessed by western blot analysis and this activation was mitigated by nuciferine. In the resting sate, NF-kB is found in the cytosol in an inhibited form due to IκB inhibitory proteins [35]. Activation of NF-κB depends on phosphorylation-induced ubiquitination of IκB proteins and leads to its nuclear translocation and the transcriptional activation of pro-inflammatory cytokines such as IL-1β and TNF-α, and chemokines such as MCP-1 [32]. Thus, it is plausible to presume that the downregulated effect of nuciferine on pro-inflammatory cytokine and chemokine expression might be through the attenuated expression of NF-κB.

The vicious cycle of reactive oxygen species generation and inflammatory response leads to renal cell degeneration through apoptosis and necrosis. In the current study, we investigated whether IRI-induced renal cell death is mediated through p53/caspase-3 dependent apoptosis pathway and the effect of nuciferine on this process. p53 induces apoptosis intrinsically by regulating the release of key apoptotic proteins [36]. This results in the destabilization of the outer mitochondrial membrane, releasing apoptogenic factors such as cytochrome c, which ends with pro-caspase-3 cleavage and activation to caspase-3 [36,37]. In this study, the IRI-induced rise in p53 expression indicates that the apoptosis was mediated through the p53 protein. Additionally, the increased expression of caspase-3 and it’s attenuation by nuciferine demonstrated by western blot in this study suggests that nuciferine attenuates IRI-induced renal dysfunction in a p53/caspase-3 dependent manner.

The involvement of autophagy in the pathological process of renal IRI has been previously reported [38–40]. In this study, western blot analysis has revealed a reduced LC3-II/LC3-I ratio with decreased maturation of LC3-I to LC3-II in response to IRI indicating the inhibition of autophagy, similar to previous reports in this condition [40–42]. This autophagy inhibition might be attributed to the role of activated caspase-3 in cleaving the autophagic protein beclin-1, which is the upstream of LC3, thereby mediating the apoptotic pathway [43]. Treatment with nuciferine significantly increased the maturation of LC3-I to LC3-II, suggesting its potential to induce autophagy.

Sustained injury and cell death lead to a fibrotic response mediated by pro-fibrotic markers such as TGF-β1 and PAI-1, leading to pro-collagen type 1 (COLA-1) accumulation in the extracellular matrix [44–46]. TGF-β1 is a master regulator of the fibrotic response and has been shown to regulate the expression of PAI-1 [44] which subsequently inhibits fibrinolysis, leading to the reduced degradation of pro-collagens, such as COLA-1 in the extracellular matrix [45]. This ultimately causes glomerulosclerosis and tubulointerstitial fibrosis [47,48]. The IRI-induced rise in the mRNA expression of TGF-β1, PAI-1 and COLA-1 in the current study was all downregulated following nuciferine treatment indicating that nuciferine has the potential to attenuate the IRI-induced tubulointerstitial fibrosis.

In addition, to its effect on the gene expression and protein level of several cytokines and factors involved in the IRI-induced alterations, nuciferine led to a significant attenuation of serum creatinine and creatinine clearance, which indicates improvements in glomerular function. Additionally, our results demonstrated reduced urinary albumin leakage as measured by 24-hour urinary albumin and albumin creatinine ratio (ACR), suggesting an improvement in the renal tubular function. This is further supported by the significant downregulation of the gene expression of two key acute injury markers, namely, KIM-1 and Lcn-2/NGAL. The latter is strongly expressed in the distal nephron [49], while KIM-1 is expressed by the proximal tubules [50]. Therefore, the current data indicated that nuciferine has an effect on different parts of the nephron leading to improvement in renal functions.

This study has some limitations. One of the limitations is the difficulty in evaluating the effect of nuciferine on the recovery of the kidney in the long-term especially in relation to the recovery of histological features due to the short-term follow-up after IRI-induced renal injury. However, the attenuation of various pro-inflammatory and pro-fibrotic cytokines and pro-apoptotic markers, including MCP-1 and cleaved caspase-3 in response to nuciferine treatment suggests that nuciferine may have a lasting effect in alleviating interstitial fibrosis following IRI. Another limitation was the lack of testing some specific renal tubular functions such as electrolyte excretion. Nonetheless, the improvements in the alterations related to the gene expression of both KIM-1 and NGAL, histological features, and ACR indicated improvements in various tubular compartments. Further studies might be required to specifically address this point. Studying the dose-response relationship in this particular condition needs also to be addressed in future research. A further limitation of this study is the lack of assessing the protein expression of all the markers studied by the gene expression analysis such as KIM-1 and ANGAL. However, the increase in the gene expression of these two markers in the G-IRI group and the attenuation of this alteration in the G-IRI/NF indicated that there were significant differences between the groups at least at the gene level. Furthermore, the observed changes in the gene expression and protein level of other markers and in the renal functional parameters and histological findings are in consistence with the changes of the gene expression of these two markers following IRI.

In the current study, we did not evaluate the changes in the gene expression and protein level of the various markers in different kidney compartments such as the cortex and medulla and this would require further research. In this study, we also did not use a pure control group. Instead, the ischemic groups were compared to the Sham group which had normal renal functions and showed normal kidney histological features similar to the normal kidneys. Further, even without the use of the pure control group, the current data has clearly shown a significant protective effect of nuciferine in the kidney post-IRI.

In conclusion, the administration of nuciferine has ameliorated the pathophysiological changes caused by the renal IRI. This effect was reflected in the partial recovery of renal functional parameters and histological features. The protective effect demonstrated in this study is attributed to its anti-inflammatory, anti-fibrotic and anti-apoptotic properties. These findings may have future clinical implications.
